# Shiga Toxin-Producing *Escherichia coli* Infection in Jönköping County, Sweden: Occurrence and Molecular Characteristics in Correlation With Clinical Symptoms and Duration of *stx* Shedding

**DOI:** 10.3389/fcimb.2018.00125

**Published:** 2018-05-01

**Authors:** Xiangning Bai, Sara Mernelius, Cecilia Jernberg, Ing-Marie Einemo, Stefan Monecke, Ralf Ehricht, Sture Löfgren, Andreas Matussek

**Affiliations:** ^1^Division of Clinical Microbiology, Department of Laboratory Medicine, Karolinska Institute, Karolinska University Hospital, Huddinge, Sweden; ^2^State Key Laboratory of Infectious Disease Prevention and Control, Chinese Center for Disease Control and Prevention, National Institute for Communicable Disease Control and Prevention, Beijing, China; ^3^Department of Laboratory Medicine, Jönköping, Sweden; ^4^The Public Health Agency of Sweden, Solna, Sweden; ^5^Department of Communicable Disease Control, Jönköping, Sweden; ^6^Abbott (Alere Technologies GmbH), Jena, Germany; ^7^Institute for Medical Microbiology and Hygiene, Technische Universität Dresden, Dresden, Germany; ^8^InfectoGnostics Research Campus, Jena, Germany; ^9^Karolinska University Laboratory, Stockholm, Sweden

**Keywords:** Shiga toxin-producing *Escherichia coli*, virulence factor, *stx* subtype, shedding, clinical symptoms

## Abstract

Shiga toxin–producing *Escherichia coli* (STEC) cause bloody diarrhea (BD), hemorrhagic colitis (HC), and even hemolytic uremic syndrome (HUS). In Nordic countries, STEC are widely spread and usually associated with gastrointestinal symptoms and HUS. The objective of this study was to investigate the occurrence of STEC in Swedish patients over 10 years of age from 2003 through 2015, and to analyze the correlation of critical STEC virulence factors with clinical symptoms and duration of *stx* shedding. Diarrheal stool samples were screened for presence of *stx* by real-time PCR. All STEC isolates were characterized by DNA microarray assay and PCR to determine serogenotypes, *stx* subtypes, and presence of intimin gene *eae* and enterohaemolysin gene *ehxA*. Multilocus sequencing typing (MLST) was used to assess phylogenetic relationships. Clinical features were collected and analyzed using data from the routine infection control measures in the county. A total of 14,550 samples were enrolled in this 12-years period study, and 175 (1.2%) stools were *stx* positive by real-time PCR. The overall incidence of STEC infection was 4.9 cases per 100,000 person-years during the project period. Seventy-five isolates, with one isolate per sample were recovered, among which 43 were from non-bloody stools, 32 from BD, and 3 out of the 75 STEC positive patients developed HUS. The presence of *stx2* in both stools and isolates were associated with BD (*p* = 0.008, *p* = 0.05), and the presence of *eae* in isolates was related to BD (*p* = 0.008). The predominant serogenotypes associated with BD were O157:H7, O26:H11, O121:H19, and O103:H2. Isolates from HUS were O104:H4 and O98: H21 serotypes. Phylogenetic analysis revealed our strains were highly diverse, and showed close relatedness to HUS-associated STEC collection strains. In conclusion, the presence of *stx2* in stool was related to BD already at the initial diagnostic procedure, thus could be used as risk predictor at an early stage. STEC isolates with *stx2* and *eae* were significantly associated with BD. The predominant serotypes associated with BD were O157:H7, O26:H11, O121:H19, and O103:H2. Nevertheless, the pathogenic potential of other serotypes and genotypes should not be neglected.

## Introduction

Shiga toxin–producing *Escherichia coli* (STEC) cause diseases ranging in severity from asymptomatic infection, non-bloody diarrhea (NBD) to bloody diarrhea (BD), hemorrhagic colitis (HC), and even life-threatening hemolytic uremic syndrome (HUS) (Tarr et al., 2005). STEC are widely spread and associated with gastrointestinal symptoms and HUS in the Nordic countries (Haugum et al., 2014; Naseer et al., 2017; Pedersen et al., 2017). The number of STEC cases reported in Sweden has been rising ever since all STEC was notified in 2004 (Peter Nolskog and Cecilia Jernberg, 2017), with the largest O157 STEC outbreak occurring on the west coast of Sweden in 2005 (Söderström et al., 2008). In 2011, a large outbreak caused by a Shiga toxin 2–producing *E. coli* serotype O104:H4 resulted in 3,816 STEC cases in Germany, and subsequently spread throughout other European countries including Sweden (Guy et al., 2012, 2013). This highlighted the clinical significance of other STEC serotypes than O157 as a great threat to public health.

The production of Shiga toxin (Stx) is the primary virulence trait responsible for STEC disease, along with the presence of an outer membrane protein intimin (*eae*), which can lead to the formation of attaching and effacing (A/E) lesions (Elliott et al., 2000). Stx can be divided into two major types (Stx1 and Stx2), several subtypes and variants for each toxin type have been described (Scheutz et al., 2012). Shiga toxin subtypes have been found to differ in receptor preference and toxin potency (Fuller et al., 2011). Strains producing subtype Stx2a, Stx2c, or Stx2d which display close sequences relatedness, are often associated with development of HC and HUS (Orth et al., 2007; Kawano et al., 2008). Epidemiological studies suggest that *stx2* along with the presence of *eae* is more often associated with severe disease and development of HUS (Orth et al., 2007). However, not all STEC infections with *stx2* and *eae* positive strains develop severe clinical outcomes. The clinical significance of STEC for humans is further determined by the production and interplay of other virulence factors, such as the plasmid-encoded enterohaemolysin (*ehxA*). The prevalence of virulence factors in clinical strains and their role in disease development are not yet fully understood.

The duration of STEC carriage is of great importance since individuals who continue to excrete the pathogen may serve as a source of infection for secondary cases. In Sweden, there is a legal requirement that all individuals with HUS-associated STEC infection must have at least one *stx* negative control sample before returning to pre-school or work at risk (Peter Nolskog and Cecilia Jernberg, 2017). Our previous investigation of STEC in Swedish children revealed that *stx* genes were excreted for several months in some patients, with a maximal duration of 256 days (Matussek et al., 2016). However, the average duration of STEC shedding in adult patients is largely unknown.

The objective of this study was to depict the prevalence and molecular characteristics of STEC in patients over 10 years of age with diarrhea in Region Jönköping County, Sweden, and illustrate the correlation of several critical STEC virulence factors (*stx* subtypes, *eae* and *ehxA*) to clinical symptoms and duration of STEC shedding.

## Materials and methods

### Ethics approval statement

Formal consent is not required, as sample and data collection is part of the routine microbiological and contact tracing work in Region Jönköping County, Sweden.

### Setting, isolation of STEC, and DNA extraction

Region Jönköping County has ~330,000 inhabitants, served by three hospitals and 46 health care centers and the clinical microbiology laboratory in Jönköping, Sweden, receives all microbiology samples in the region. The current study comprised all routine diarrheal stool samples where STEC analysis was requested by the clinician. In addition, STEC analysis was performed on stool samples submitted for routine bacterial culture where BD and/or HUS was mentioned on the referral as well as from individuals involved in contact tracing around an index case. All samples included were evaluated for the presence of *stx* by real-time PCR on suspensions of overnight cultures on blood agar plates at the clinical microbiology laboratory. Primers (MWG-Biotech, Ebersberg, Germany) and probes (TIB MOLBIOL, Berlin, Germany) used in the current study for detection of *stx1* and *stx2* were described previously (Bellin et al., 2001; Table S1). Real-time PCR was performed on LightCycler® 480 Instrument II (Roche Diagnostics GmbH, Mannheim, Germany). The amplification program included an initial denaturation step at 95°C for 120 s and 45 cycles of denaturation at 95°C for 1 s, annealing at 55°C for 5 s (reached with a touchdown from 60°C over the course of the first five cycles), and extension at 72°C for 20 s. *E. coli* EDL 933 was used as a positive control strain and nuclease-free water was used as negative control for real-time PCR method. PCR positive samples were then sent to the Karolinska University Laboratory, Stockholm, Sweden for confirmation and isolation of STEC strains on Sorbitol-MacConkey (SMAC) plates according to methods previously described (Svenungsson et al., 2000). Patients were sampled weekly until they were *stx*-PCR negative, and the duration of *stx* shedding was defined as the time from the first positive sample to the first negative sample (Matussek et al., 2016). Bacterial DNA was extracted from overnight cultures with EZ1 DNA Tissue Kit (Qiagen, Hilden, Germany) on EZ1 instrument (Qiagen).

### Clinical data collection

Clinical data was collected from all patients who tested positive for *stx* by PCR over 10 years of age in Jönköping county from April 2003 through January 2015 through a questionnaire and by reviewing medical records as part of the routine infection control measures in the county. Clinical manifestations included were diarrhea, BD, abdominal pain, vomiting, fever and HUS. HUS was characterized by three primary symptoms: thrombocytopenia, microangiopathic hemolytic anemia, and acute renal injury according to Bryan et al. (2015).

### Serotyping, *stx* subtyping and detection of *eae* and *ehxA*

In Sweden, all STEC isolates are submitted to The Public Health Agency of Sweden for confirmation and further typing as part of the national microbial surveillance program. This includes STEC O-type serogrouping by agglutination in micro titer plates using antisera (SSI Diagnostica, Copenhagen, Denmark).

All STEC isolates were further subjected to microarray based assays with the *E. coli* SeroGenoTyping AS-1 Kit, ShigaToxType AS-2 Kit (Alere Technologies GmbH, Germany) to determine the serogenotype (O:H) (Geue et al., 2014a) and *stx* allele/subtype (Scheutz et al., 2012; Geue et al., 2014b) according to manufacturer's instructions (http://alere-technologies.com/en/products/lab-solutions/e-coli/e-coli-serogenotyping-kit.html, http://alere-technologies.com/en/products/lab-solutions/shigatoxin.html). Detection of *eae* and *ehxA* was done by PCR (Bai et al., 2016).

### MLST

Multilocus sequence typing (MLST) was used to characterize phylogenetic relationships of strains. Defined fragments of the seven housekeeping genes (i.e., *adk, icd, fumC, recA, mdh, gyrB*, and *purA*) were amplified and sequenced according to the *E. coli* MLST website (https://enterobase.warwick.ac.uk/species/ecoli/allele_st_search). Sequences types (STs) were assigned based on the allelic profile of the seven housekeeping genes. STs of isolates from this study were compared with HUS-associated enterohemorrhagic *E. coli* (HUSEC) collection strains (www.ehec.org) (Mellmann et al., 2008). A minimum spanning tree (MST) based on these STs was generated using BioNumerics version 7.6 (Applied Maths NV, Sint-Martens-Latem, Belgium).

### Statistical analyses

χ2 and Fisher's exact test were done for comparing categorical data using IBM® SPSS® Statistics 24.0 (IBM, US). Multiple testing correction by using the Benjamini-Hochberg method was conducted to correct the individual *p*-value for each gene. *p* < 0.05 was considered statistically significant.

## Results

### Patient data and the prevalence of STEC

A total of 14,550 specimens were enrolled in this 12-years period investigation. 175 (1.2%) stools were *stx* positive by real-time PCR on suspensions of overnight cultures on blood agar plates, and 85 of them were *eae* positive. The overall incidence of STEC infection was 4.9 cases per 100,000 person-years, with the annual incidence varying from 3.70 to 8.03 cases per 100,000 persons per year (Table 1). The presence of *stx2* in stool samples was associated with BD (*p* = 0.008), while a higher positive rate of *stx1*+*stx2* was detected in non-bloody stool (NBS) samples (*p* = 0.008; Table 2). Seventy-five *stx* positive stools yielded isolates on SMAC plates, with one isolate from one sample each, giving a culture positive rate of 42.9%. STEC patient data and clinical symptoms are presented in Table 3. The presence of *stx2*-only in isolates was associated with BD (*p* = 0.05; Table 2).

**Table 1:** STEC incidence in adults over 10 years of age from April 2003 to January 2015, Jönköping county, Sweden.

**Sampling year**	**Population(>10 years old)**	**STEC cases**	**Annual incidence rate per 100,000 persons**	**No. of STEC isolates**	**Culture positive rate (%)**
2003	291,922	12	4.11	7	58.33
2004	293,045	14	4.78	8	57.14
2005	294,097	17	5.78	6	35.29
2006	295,187	11	3.73	4	36.36
2007	296,628	14	4.71	7	50.00
2008	297,835	11	3.70	3	27.27
2009	298,028	14	4.70	4	28.57
2010	298,272	14	4.70	2	14.29
2011	298,650	24	8.03	11	45.83
2012	299,367	14	4.68	10	71.43
2013	300,898	15	4.98	8	53.33
2014	303,195	15	4.95	5	33.33
Total	3567,124	175	4.91	75	42.86

**Table 2:** Prevalence of *stx* and *eae* detected on stools and isolates in correlation with bloody diarrhea (BD) and non-bloody stools (NBS).

**Virulence genes**	**No. of stools (%)**		***p*-value**	**No. of isolates (%)**		***p*-value**
	**BD (*n* = 53)**	**NBS (*n* = 122)**		**BD (*n* = 32)**	**NBS (*n* = 43)**	
*stx1*-only	14(26.4)	37(30.3)	0.601	7(21.9)	14(32.6)	0.601
*stx2*-only	26(49.1)	15(12.3)	0.008^*^	17(53.1)	10(23.3)	0.05^*^
*stx1*+*stx2*	13(24.5)	70(57.4)	0.008^*^	8(25.0)	19(44.2)	0.271
*eae*	32(60.4)	53(43.4)	0.163	22(68.8)	13(30.2)	0.008^*^

* *Statistically significant difference*.

**Table 3:** Patient data and clinical symptoms for all STEC infections yielding isolates.

	**All cases**	**BD**	**NBS**
No. (%)	75	32 (43)	43 (57)
Median age of patients	41 (10–87)	49 (15–87)	38 (10–82)
Median length of carriage, in days	17 (0–294)	17 (7–197)	18 (0–294)
Aquired bacteria abroad (%)	19 (25.3)	7 (21.9)	12 (27.9)
Diarrhea (%)	54 (72.0)	32 (100)	22 (51.2)
Abdominal pain (%)	39 (52.0)	25 (78.1)	14 (32.6)
Fever (%)	10 (13.3)	2 (6.3)	8 (18.6)
Vomiting (%)	3 (4.0)	0 (0)	3 (7.0)
HUS (%)	3 (4)	2 (6.3)	1 (2.3)

### *stx* subtypes and presence of *eae, ehxA*

Overall, two *stx1* subtypes (i.e., *stx1a* and *stx1c*) and six *stx2* subtypes (i.e., *stx2a, stx2b, stx2c, stx2d, stx2e*, and *stx2g*) were detected, resulting in a total of 16 different *stx* subtype combinations (Table 4 and Table S2). BD cases were caused by *stx1a-*only, *stx1a*+*stx2a, stx1a*+*stx2c, stx1c*+*stx2b, stx2a*-only, *stx2a*+*stx2c, stx2b*-only, and *stx2g-*only positive isolates. Two of the three HUS isolates carried *stx2a*-only, and one HUS isolate possessed *stx1a*-only. *stx2a* only, *stx2a*+*stx2c, stx1a*+*stx2a*, and *stx1a*+*stx2c* were more often detected in isolates from BD cases, while no statistically significant difference was found (Table S2). In total, 35 isolates harbored *eae*, and 59 carried *ehxA*. Interestingly, almost all *eae* positive isolates, with one exception, carried *ehxA*. The presence of *eae* in isolates was found to be associated with BD (*p* = 0.008; Table 2). Notably, only one of three HUS isolates carried the *ehxA* gene, and none of them harbored *eae*.

**Table 4:** Sequence types, serotypes, virulence genes in 75 STEC isolates and associated-clinical symptoms^a^.

**ST**	**No**.	**Serotypes**	**Virulence genes/*stx* subtypes**	**Clinical symptoms**
11	15	O157:H7 (15)	*stx2a*+*stx2c* (11), *stx1a*+*stx2a* (1), *stx1a*+*stx2c* (3), *eae* (15), *ehxA* (15)	BD (11), NBS (4)
21	9	O26:H11 (9)	*stx1a* (8), *stx2a* (1), *eae* (3), *ehxA* (9)	BD (6), NBS (3)
655	6	O121:H19 (6)	*stx2a* (5), *stx1a*+*stx2a* (1), *eae* (6), *ehxA* (5)	BD (3), NBS (3)
17	5	O103:H2 (5)	*stx1a* (5), *eae* (5), *ehxA* (5)	BD (2), NBS (3)
678	4	O104:H4 (4)	*stx2a* (4)	HUS (2), BD (1), NBS (1)
442	3	O146:H21 (1), O91:H21 (2)	*stx2b* (1), *stx2d* (2), *ehxA* (3)	BD (1), NBS (2)
504	3	O117:H7 (2), O156:H7 (1)	*stx1a* (3)	NBS (3)
10	2	O113:H4 (1), O4:H16 (1)	*stx1c* (1), *stx2d* (1), *ehxA* (1)	NBS (2)
13	2	O117:H8 (2)	*stx1c* (2), *ehxA* (2)	NBS (2)
33	2	O91:H14 (2)	*stx1a*+*stx2b* (2), *ehxA* (2)	NBS (2)
1724	2	O150:H10 (2)	*stx2b* (2), *ehxA* (2)	NBS (2)
25	1	O128ab:H2	*stx1c*+*stx2b, ehxA*	NBS
58	1	O126:H20	*stx2a, ehxA*	NBS
119	1	O165:H25	*stx2a*+*stx2c, eae, ehxA*	BD
137	1	O145:H28	*stx2a, eae, ehxA*	NBS
301	1	O180:H2	*stx2a, eae, ehxA*	NBS
306	1	O98:H21	*stx1a, ehxA*	HUS
325	1	O15:H16	*stx2g*	NBS
329	1	O136:H12	*stx2a, ehxA*	NBS
388	1	O112ab:H2	*stx2b*+*stx2d*	NBS
410	1	O8:H9	*stx2c*	NBS
447	1	O5:H19	*stx1c, ehxA*	NBS
657	1	O183:H18	*stx1a*+*stx2a, ehxA*	BD
658	1	O103:H28	*stx1a*+*stx2a, eae, ehxA*	BD
679	1	O163:H19	*stx1a*+*stx2d, ehxA*	NBS
738	1	O146:H28	*stx2b*	NBS
811	1	O128ac:H2	*stx1c*+*stx2b*	BD
1494	1	O9a:H21	*stx2e*	NBS
1804	1	O157:H7	*stx1a*+*stx2c, eae, ehxA*	BD
2388	1	O15:H27	*stx1c*+*stx2b*	NBS
3101	1	O78:H4	*stx1c*+*stx2b, ehxA*	NBS
N1^b^	1	O91:H21	*stx2c*+*stx2d, eae, ehxA*	NBS
N2^b^	1	O187:H28	*stx2g, ehxA*	BD

a *The number of isolates is indicated in parentheses if it contains more than one isolate*.

b *Two new STs assigned in this study*.

### Serotypes

Thirty-four distinct serotypes were found in 75 isolates, which comprised of 29 distinct O serogroups and 17 H types. The most prevalent serotype was O157:H7 (*n* = 16), followed by O26:H11 (*n* = 9), O121:H19 (*n* = 6), O103:H2 (*n* = 5), O104:H4 (*n* = 4), and O91:H21 (*n* = 3) (Table 4). Serotypes causing 32 BD cases were O157:H7(*n* = 12), O26:H11(*n* = 6), O121:H19 (*n* = 3), O104:H4 (*n* = 3), O103:H2 (*n* = 2), O103:H28 (*n* = 1), O128ac:H2 (*n* = 1), O146:H21(*n* = 1), O165:H25 (*n* = 1), O183:H18 (*n* = 1), O187:H28 (*n* = 1). Isolates from individuals that developed HUS were assigned to O104:H4 (*n* = 2), O98:H21 (*n* = 1), which harbored *stx2a*-only and *stx1a*-only subtype, respectively.

### Duration of *stx* shedding

Data on duration of *stx* shedding was available for 39 (52%) patients, ranging from 0 to 294 days. The median length of carriage was 17 days (Table 3), which was used to separate short (<2.5 weeks) and long (≥ 2.5 weeks) carriage. No statistically significant difference was found between the presence of genes tested and long duration of *stx* shedding in this study (Table S3).

### MLST

**Figure 1:**
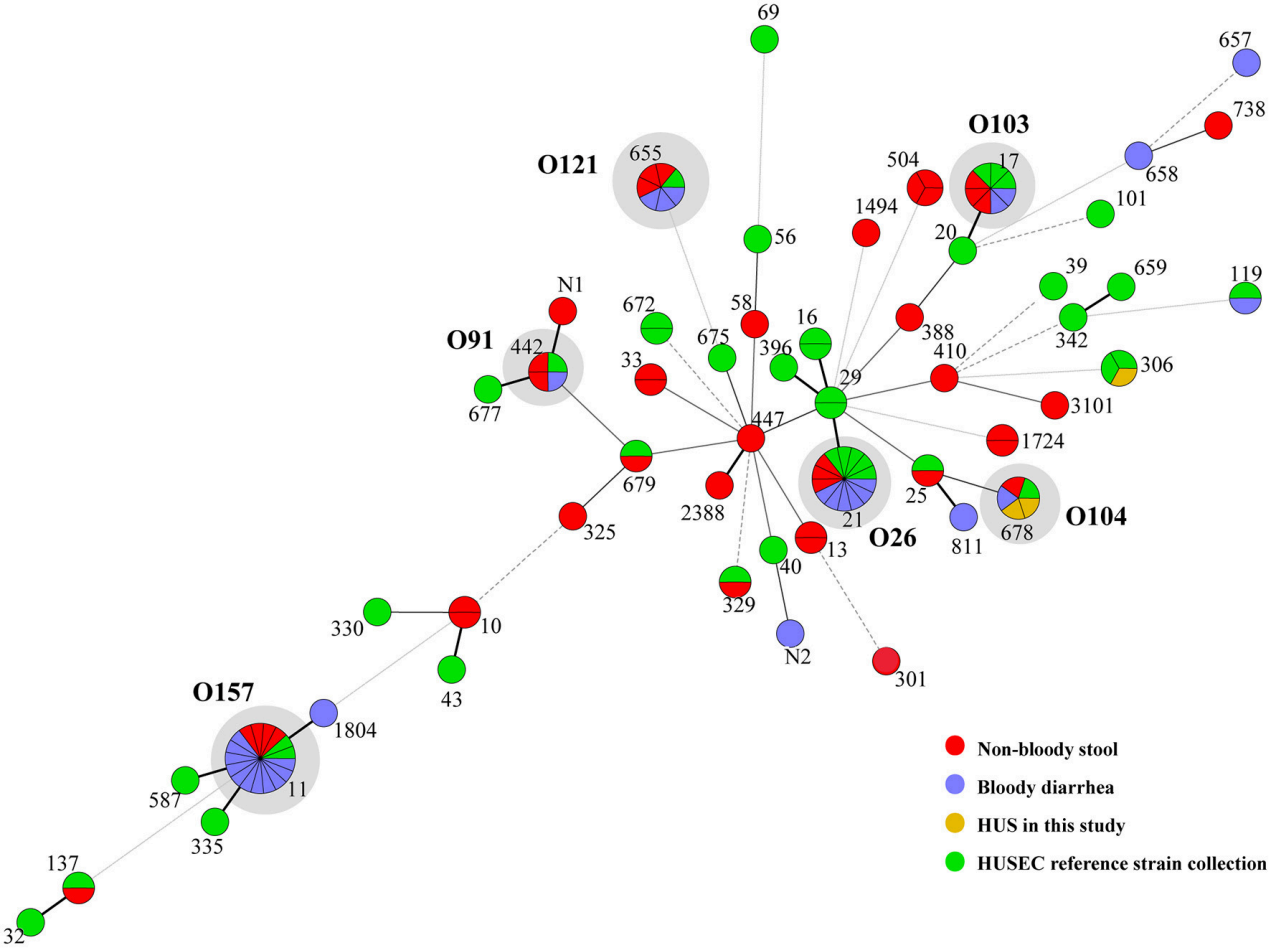
Minimum spanning tree of 33 STs in this study and 31 STs from HUSEC collection. Each circle represents an ST with the size being proportional to the number of isolates. The colors for the slices of the pie represent the sources of isolates (see labeling in the lower right corner). The predominant STs comprising of strains in this study and HUSEC reference collection are indicated in gray shadows, with serogroups demonstrated aside.

Thirty-three sequence types (STs) were obtained from 75 STEC isolates, including two novel STs (Table 4). It's noteworthy that isolates belonging to the same serotype (O157:H7, O26:H11, O121:H19, O103:H2, and O104:H4) were assigned as same ST (i.e., ST11, ST21, ST655, ST17, and ST678, respectively). 12 STs from BD cases and 26 STs from NBS in this study were scattered throughout the phylogenetic tree (Figure 1). Except the two HUS associated STs (ST678 and ST306), we found several STs from both NBS and BD in this study shared same STs (ST11, ST17, ST21, ST442, ST655, and ST679) or showed close relationship with HUSEC collection strains.

## Discussion

The incidence of reported human STEC cases varies geographically in Sweden. For example, 30.3 cases per 100,000 inhabitants were reported in Halland in 2016, while the reported incidence in Örebro County was 0 (Peter Nolskog and Cecilia Jernberg, 2017). In this report, the annual STEC incidence in individuals over 10 years of age in Jönköping county varied from 3.70 to 8.03 cases per 100,000 person-years during the project period. Sweden shows higher STEC incidence when compared with other Nordic countries and non-European countries (Pedersen et al., 2017; Vasant et al., 2017). The various STEC incidence might be effected by different local sampling and analysis routines (Peter Nolskog and Cecilia Jernberg, 2017). STEC isolation was failed in 57.1% of *stx* positive samples on SMAC agar in this study, including one HUS case, which could result in underestimation of clinical significance of emergent non-O157 seropathotypes. The low isolation rate might be due to shortcomings in current STEC screening protocols, for instance, the SMAC agar, which has been widely used for O157 isolation, is not sufficiently discriminatory to detect emergent non-O157 serotypes (Thomas et al., 2017). On the other hand, the transport conditions and time delay to culture in the reference laboratory, lack of culture enrichment broth in the current study could also be taken into account. Further studies are required to improve the diagnostic algorithm applied on clinical samples in combination with PCR diagnostics and culture based molecular serotyping as reported previously (de Boer et al., 2015). Remarkably, with increasing availability of whole-genome sequences of *E. coli* strains genome-scale metabolic models, whole genome sequencing (WGS) will contribute to greater understanding of STEC, aid to the development of improved culture-based detection methods in routine STEC diagnosis and provide a fast and accurate alternative to conventional identification and typing techniques (Sadiq et al., 2014; Lindsey et al., 2016; Parsons et al., 2016; Newell and La Ragione, 2018).

In this report, we found that *stx2* was related to BD already at the initial PCR diagnosis on stools. Further characterization of STEC isolates enhanced that isolates harboring *stx2* and *eae* were significantly associated with BD, therefore could be used as key genetic markers for risk assessment. It's noteworthy that we did not found association between *stx1/stx2* subtypes and clinical symptoms, which is understandable as the number of isolates assigned as each subtype is limited in this study. Remarkably, three HUS isolates were all *eae* negative, no correlation between *eae*/*stx2* subtypes and HUS was observed, which was also in agreement with a recent report from Denmark (Pedersen et al., 2017). This might partly be due to the low number of HUS cases in our study, and it is noteworthy that the two O104:H4 HUS isolates were from Swedish patients that visited Germany during the O104:H4 outbreak in 2011, exhibiting same serotype, *stx2a* subtype and absence of *eae* with the outbreak strain, thus they might be the same strain. In the O104:H4 outbreak strain, instead of intimin (*eae*), it's the aggregative adherence encoded by pAA plasmid genes (*aggA, aggR*) that anchored the bacterium to the intestinal mucosa (Bielaszewska et al., 2011; Navarro-Garcia, 2014). The absence of *eae* in HUS and BD cases implied that other adherence factors might contribute to severe disease outcomes. It's noteworthy that one HUS patient was infected with *stx1a*-only isolate, the same genotype was also observed in HUS case in Germany, suggesting that *stx2* and *eae* could not reliably differentiate between HUS-associated and non-HUS-associated STEC strains. Hence, the potential of *stx1* and other *stx2* subtypes in development of HUS should not be neglected.

The predominant serotypes associated with BD cases in this study were in accordance with our previous survey in children (Matussek et al., 2016), and also similar to that reported in Norway (Naseer et al., 2017). Except O104:H4 isolates, one O98:H21 isolate from a HUS case, showed same sequence type (ST306), O serogroup and *stx1* type as a HUSEC strain in Germany, highlighting the potential risk of non-O157 seropathotypes that were not predominantly reported. Interestingly, there is a good concordance observed between serotype and ST in this study. Isolates of same serotype exhibited higher tendency to be assigned as same ST, especially for predominant serotypes (O157:H7, O26:H11, O121:H19, O103:H2, and O104:H4). Thus, ST could be used to help molecularly assign a serotype in STEC genotyping. Moreover, these predominant STs were more often detected in BD cases, and all were found in HUSEC collection in Germany (Mellmann et al., 2008), implying that some STs showed higher pathogenic potential, could be used as high risk prediction in molecular typing.

The present study was confined to several limitations, for example, the low STEC isolation rate, which might result in the underestimation of clinical significance of non-O157 serotypes, and also insufficient association of the presence of virulence genes with symptomatology; limited number of virulence genes detected, HUS isolates and duration of shedding data in this study, which might prevent an adequate statistical association analysis between diverse genes/genotypes and clinical symptoms as well as duration of *stx* shedding. Hence, enhancement of STEC diagnosis of various serotypes in clinical samples, and whole genome-based analysis of molecular traits in correlation with clinical symptoms and duration of *stx* shedding is further needed.

In conclusion, here we report a large scale STEC investigation in Swedish patients over a 12-years period. The overall incidence of STEC infection was 4.9 cases per 100,000 person-years, 1.2% samples were *stx* PCR positive in investigated cases. By further characterization on serotypes, key virulence genes, *stx* subtypes, and MLST, we found the presence of *stx2* in stool was related to BD already at the initial PCR diagnostic procedure performed directly on stool suspensions, thus could be used as risk predictor at an early stage. Further, isolates with *stx2* and *eae* were significantly associated with BD. Isolates with high virulent *stx2a*/*stx2c* subtypes and serotypes (O157:H7, O26:H11, O121:H19, and O103:H2) are more prevalent in BD case, while strains with *stx1a* only and non-predominant serotype was associated with HUS. Thus, the pathogenic potential of other serotypes and genotypes should not be neglected.

## Author contributions

AM and XB designed the experiments. SMe, CJ, SMo, and RE performed the experiments. I-ME and SMe collected clinical data. SMo and SL contributed analysis. XB and AM wrote the paper. SMe, CJ, SMo, RE, and SL polished the paper.

### Conflict of interest statement

The authors declare that the research was conducted in the absence of any commercial or financial relationships that could be construed as a potential conflict of interest.
