# Supplementary material for: Shiga Toxin-Producing Escherichia coli Infection in Jönköping County, Sweden: Occurrence and Molecular Characteristics in Correlation With Clinical Symptoms and Duration of stx Shedding
Source: Front Cell Infect Microbiol. 2018 May 1;8:125. doi: 10.3389/fcimb.2018.00125 (PMC5939558; doi:10.3389/fcimb.2018.00125)
Supplement: Supplementary file 1 [file Table_1.DOCX]

Supplementary Material

**Shiga Toxin-Producing *Escherichia coli* Infection in Jönköping County, Sweden: Occurrence and Molecular Characteristics in Correlation with Clinical Symptoms and Duration of *stx* Shedding**

**Xiangning Bai, Sara Mernelius, Cecilia Jernberg, Ing-Marie Einemo, Stefan Monecke, Ralf Ehricht, Sture Löfgren, Andreas Matussek**

***Correspondence:** Andreas Matussek: andreas.matussek@ki.se

**Supplementary Tables**

**TABLE S1. Nucleotide sequences of primers and probes used in this study**

| **Primer or probe** | **Sequence (5′→3′)** | **Tm (°C)** |
| --- | --- | --- |
| Primers |  |  |
| *stx1* | AGT CGT ACG GGG ATG CAG ATA AAT / | 56.9 |
|  | CCG GAC ACA TAG AAG GAA ACT CAT | 55.3 |
| *stx2* | TTC CGG AAT GCA AAT CAG TC / | 52.5 |
|  | CGA TAC TCC GGA AGC ACA TTG | 54.6 |
| Probes |  |  |
| *stx1* | CTG TCA CAG TAA CAA ACC GTA ACA TCG CTC-X / | 65.5 |
|  | LC-TGC CAC AGA CTG CGT CAG TGA GGT-ph | 67.5 |
| *stx2* | MAG AGC AGT TCT GCG TTT TGT CAC TGT CA-X / | 65.0 |
|  | LC-AGC AGA AGC CTT ACG CTT CAG GC-ph | 63.3 |

**TABLE S2. Prevalence of different *stx* subtype combinations and *ehxA* in STEC isolates from BD and NBS**

| **Genes** | **No. of isolates** | **BD (%)** |  | **NBS (%)** | **p-value** |
| --- | --- | --- | --- | --- | --- |
| *stx1a* | 17 | 7^a^ (21.9) |  | 10 (23.2) | 0.888 |
| stx2a | 14 | 7^b^ (21.9) |  | 7 (16.3) | 0.538 |
| *stx2a*+*stx2c* | 12 | 8 (25) |  | 4 (9.3) | 0.067 |
| *stx1a*+*stx2a* | 4 | 3 (9.4) |  | 1 (2.3) | 0.307 |
| *stx1c*+*stx2b* | 4 | 1 (3.1) |  | 3 (7.0) | 0.632 |
| *stx2b* | 4 | 1 (3.1) |  | 3 (7.0) | 0.632 |
| *stx1a*+*stx2c* | 4 | 4 (12.5) |  | 0 (0) | 0.51 |
| *stx1c* | 4 | 0 (0) |  | 4 (9.3) | 0.131 |
| *stx2g* | 2 | 1 (3.1) |  | 1 (2.3) | 1.000 |
| *stx1a*+*stx2b* | 2 | 0 (0) |  | 2 (4.7) | 0.504 |
| *stx1a*+*stx2d* | 1 | 0 (0) |  | 1 (2.3) | 1.000 |
| *stx2b*+*stx2d* | 1 | 0 (0) |  | 1 (2.3) | 1.000 |
| *stx2c* | 1 | 0 (0) |  | 1 (2.3) | 1.000 |
| *stx2c*+*stx2d* | 1 | 0 (0) |  | 1 (2.3) | 1.000 |
| *stx2d* | 3 | 0 (0) |  | 3 (7.0) | 0.256 |
| *stx2e* | 1 | 0 (0) |  | 1 (2.3) | 1.000 |
| *ehxA* | 59 | 28 (87.5) |  | 31 (72.1) | 0.107 |

^a^ One *stx1a* isolate was from HUS

^b^ Two *stx2a* isolates were from HUS

**TABLE S3. Association between virulence genes and duration of *stx* shedding**

| **Virulent genes/subtypes** | **Duration of *stx* shedding (weeks)** | |  |
| --- | --- | --- | --- |
|  | **>2.5 (n=19)** | **<2.5 (n=20)** | **p-value** |
| *stx1a* | 7 | 4 | 0.243 |
| *stx1a*+*stx2a* | 1 | 2 | 1.000 |
| *5stx1a*+*stx2b* | 1 | 0 | 0.487 |
| *stx1a*+*stx2c* | 0 | 2 | 0.487 |
| *stx1a*+*stx2d* | 0 | 1 | 1.000 |
| *stx1c*+*stx2b* | 2 | 0 | 0.231 |
| *stx2a* | 5 | 2 | 0.235 |
| *stx2a*+*stx2c* | 0 | 5 | 0.380 |
| *stx2b* | 1 | 0 | 1.000 |
| *stx2b*+*stx2d* | 1 | 0 | 1.000 |
| *stx2c* | 1 | 0 | 1.000 |
| *stx2c*+*stx2d* | 0 | 1 | 1.000 |
| *stx2d* | 0 | 2 | 0.487 |
| *stx2g* | 0 | 1 | 1.000 |
| *eae* | 6 | 14 | 0.260 |
| *ehxA* | 13 | 18 | 0.127 |
